# Comparison of Annoyance from Railway Noise and Railway Vibration

**DOI:** 10.3390/ijerph14070805

**Published:** 2017-07-19

**Authors:** Mikael Ögren, Anita Gidlöf-Gunnarsson, Michael Smith, Sara Gustavsson, Kerstin Persson Waye

**Affiliations:** 1 Occupational and Environmental Medicine, Institute of Medicine, Sahlgrenska Academy, University of Gothenburg, 40530 Gothenburg, Sweden; michael.smith@amm.gu.se (M.S.); kerstin.persson.waye@amm.gu.se (K.P.W.); 2 Department of Occupational and Environmental Medicine, Faculty of Medicine and Health, Örebro University, 70281 Örebro, Sweden; anita.gidlof-gunnarsson@regionorebrolan.se; 3 Health Metrics Unit, Institute of Medicine, Sahlgrenska Academy, University of Gothenburg, 40530 Gothenburg, Sweden; sara.gustavsson@gu.se

**Keywords:** railway noise, railway vibration, annoyance

## Abstract

The aim of this study is to compare vibration exposure to noise exposure from railway traffic in terms of equal annoyance, i.e., to determine when a certain noise level is equally annoying as a corresponding vibration velocity. Based on questionnaire data from the Train Vibration and Noise Effects (TVANE) research project from residential areas exposed to railway noise and vibration, the dose response relationship for annoyance was estimated. By comparing the relationships between exposure and annoyance for areas both with and without significant vibration exposure, the noise levels and vibration velocities that had an equal probability of causing annoyance was determined using logistic regression. The comparison gives a continuous mapping between vibration velocity in the ground and a corresponding noise level at the facade that are equally annoying. For equivalent noise level at the facade compared to maximum weighted vibration velocity in the ground the probability of annoyance is approximately 20% for 59 dB or 0.48 mm/s, and about 40% for 63 dB or 0.98 mm/s.

## 1. Introduction

Railway traffic is expected to increase in Europe, both for freight and passenger transport [1]. Increasing railway traffic will lead to more noise and vibration exposure for those living close to railways. Perceivable vibration in buildings can be generated by railway vehicles moving along the rail. The moving load of the train together with varying stiffness and irregularities along the rail generate ground waves that interact with the building foundation. The vibrations are more prominent for soft ground types and for heavy freight trains with high axle loads.

Recently, the Swedish Transport Administration estimated the number of people exposed to vibration along Swedish railways using an empirical calculation approach based on ground type data, distance to railway and railway traffic [2]. Approximately 54,000 persons are exposed to a maximum weighted velocity of 0.4 mm/s or above. The corresponding number for 1.0 mm/s is 14,000 persons. For noise from railways, the official estimation from the Swedish Environmental Protection Agency gives the number of exposed above 55 dB equivalent level as approximately 230,000 persons [3].

Noise from railway traffic is measured and evaluated using similar methods globally, but for vibration there exist many different measurement and evaluation methods, and vibration exposure measured in different countries is difficult to compare. An overview and some guidelines for comparing results was published by the European research project CargoVibes [4]. In the present study the Swedish standard for measuring vibration was used [5], where the vibration velocity is measured on the floor and weighted using a weighting factor identical to Wm from ISO 2631-1 [6], and then time weighting SLOW (i.e., 1 s) is applied. The same procedure is applied to all three directions (parallel and perpendicular to the railway and vertical) and the maximum value is selected.

Humans perceive vibration through several different pathways, and the sensitivity is dependent on many things, among others vibration direction, amplitude, frequency, body position, contact points between the vibrating surface and the human body and muscle tension [7]. For railway noise the pathway is via the auditory system, and for vibration it is manly via the somatosensory system. The interaction between auditory and vibration stimuli is not well known at the frequency range of interest here (5–10 Hz), but research on their impact on sleep shows that there seems to be an additive effect for cortical arousals and sleep stage changes [8,9]. For higher frequencies and higher sound pressure levels (>100 dB) the noise will induce vibration on the body surface [10].

To assess disturbance, discomfort and interference of activities or mood by noise and vibration, noise annoyance is measured in an exposed population. The measurement is standardized in [11]. Dose-response curves for annoyance have existed for some time for railway noise [12] and have more recently been developed for railway vibration [13]. This paper investigates at what vibration velocity the annoyance from vibration equates to the annoyance from noise. An important complication is the interdependence of annoyance, whereby the annoyance due to noise may be influenced by the presence of vibration [14]. This study investigates annoyance from railway noise and vibration based on questionnaire data in two areas.

## 2. Materials and Methods

### 2.1. Study Population

The questionnaire data were collected in 2007 within the Train Vibration and Noise Effects (TVANE) project [14]. Two study sites in Sweden (area 1, noise only, Töreboda and Falköping) were selected in areas with relatively intense railway traffic and no vibrations from railway traffic. Two separate study sites (area 2, noise and vibration, Alingsås (2A) and Kungsbacka (2B)) were selected in areas with approximately the same number of trains as in area 1 but where the trains induced strong vibrations in the ground and the dwellings. Three of the study sites (Töreboda, Falköping, and Alingsås (2A)) were situated at the railway line “Västra Stambanan” between Gothenburg and Stockholm, and the fourth study site (Kungsbacka (2B)) was situated at the railway line “Västkustbanan” south of Gothenburg. The total numbers of daily and night time train passages (and freight train passages) are presented in Table 1.

The areas were also selected so that other noise sources did not influence the results. Highways and major roads were avoided, but local road traffic at slow speeds was present in all areas.

Within the selected areas all individuals in each household between 18–75 years of age and who had lived at the address for at least six months were chosen for the study. The study comprised in total 862 participants and the response rate for area 1 was 49%, for area 2A 54%, and for area 2B 53%. The mean age was highest in area 2B (54.8%) followed by area 2A (49.9%) and area 1 (48.0%). Somewhat more men participated in area 1 (56% men), but the reverse was the case in area 2A and area 2B (53% and 54% women, respectively). A higher proportion of the respondents in area 2B (89%) were married or de facto co-habiting than in the two other areas (59% and 64%). A majority of the respondents in the three areas were employed or had their own company (64–72%), and the rest had different status such as studying, retirement (early retirement, sickness or old-age pensioner), unemployed, or were on sick or parental leave. In all three areas, about the same proportion of the respondents (28–32%) had a high level of education (≥3 years at university). Sensitivity to sound/noise was lower in area 1 (20%) than in the two other areas (30%). The average time of residence was higher in area 2A (16.1 years) than in area 1 (10.2 years) and in area 2B (12.1 years). All residents in area 2B lived in detached houses, in areas 1 and 2A 50% lived in detached houses and 50% in apartment buildings.

### 2.2. Survey

Annoyance and other health effects due to traffic noise exposure were evaluated by questionnaires. The format is similar to the ones previously used in larger epidemiological studies of noise annoyance in Sweden [1,2] and included 50 questions in total. The questionnaire was sent with an introductory letter to selected persons (all persons aged 18 to 75 years) in April 2007 (area 1) and in November 2007 (area 2). The letter described the study as an investigation of the general living environment, human health and well-being. Two reminder letters were sent out with 10 days intervals to those who not responded to the questionnaire. The first reminder consisted only of a letter while the other consisted of the reminder letter and a new questionnaire.

Both general annoyance caused by railway noise and general annoyance caused by railway vibrations were evaluated with an 11-point numerical scale (0–10 with verbal endpoints “not at all” and “extremely”) according to the ISO specification of noise annoyance scales [11]. The question about noise was phrased as follows: “Thinking about the last 12 months or so, when you are here at home, how much does noise from a railway annoy or disturb you”. The question about vibration was phrased as follows: “Thinking about the last 12 months or so, when you are here at home, how much does vibrations from a railway annoy or disturb you”.

A 6-point verbal scale with an annoyance cutoff of 3 was also used to evaluate annoyance, but the results were very similar regardless of which question which was used to define annoyance. We therefore only present results for the 11-point scale. The Spearman correlation between the question responses was 0.68 for noise and 0.85 for vibration.

### 2.3. Noise Exposure

**Figure 1:**
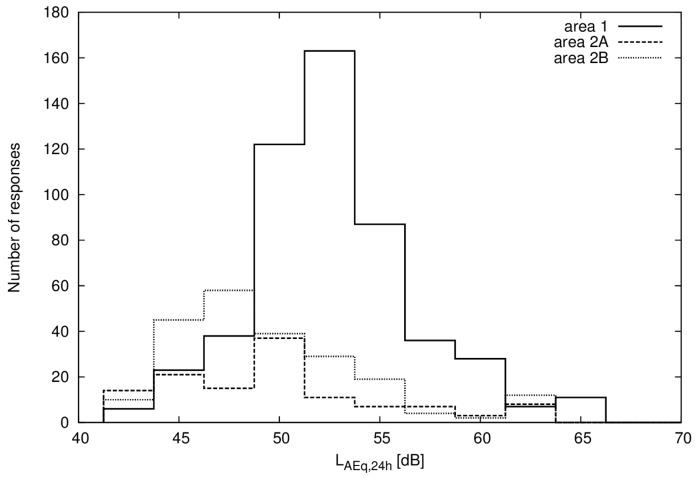
Histogram of number of responses in 2.5 dB intervals of equivalent noise level for area 1 (no vibration) and area 2 (noise and vibration).

Equivalent and maximum A-weighted noise levels were calculated for all respondents in the questionnaire surveys using the standardized Nordic method [15], which uses the distance from the railway, screening by buildings, terrain and noise barriers together with traffic data for the different train types and speeds to estimate the free field noise levels. The method is the official method for the prediction of railway traffic noise in Sweden. The number of respondents and range of calculated noise levels are presented in Table 2 and Figure 1.

### 2.4. Vibration Exposure

The vibration velocity was estimated from a total of 16 measurements of both ground and indoor vibration in Alingsås (2A, 10 measurements) and Kungsbacka (2B, 6 measurements). All measurements were performed for one building using one tri-axial accelerometer indoor on the floor, one vertical accelerometer on the ground outside the building and one reference position close to the railway. The vibration was measured for at least 24 h and the results were analyzed and weighted according to the applicable Swedish standard [5], which gives a maximum weighted vibration velocity. For buildings with more than one floor, the measurement position was on the top floor, and measurements were never performed in the basement. A few shorter sample measurements were also performed in the noise only area, which gave low vibration velocities, often below the detection threshold (0.05 mm/s) but at most 5 times lower than the levels in the ground in the vibration sensitive areas at the same distance from the railway.

For areas 2A and 2B the ground vibration velocity was estimated for all buildings not being measured by fitting a simple two parameter model to the measured data. The parameters included in the estimation was area (2A or 2B) and distance to railway, and the distance dependence was assumed to follow calculated decay functions for soft soils from [16]. A report describing all of the measurements and the method used to estimate the vibration velocity for all buildings is available in Swedish [17].

**Figure 2:**
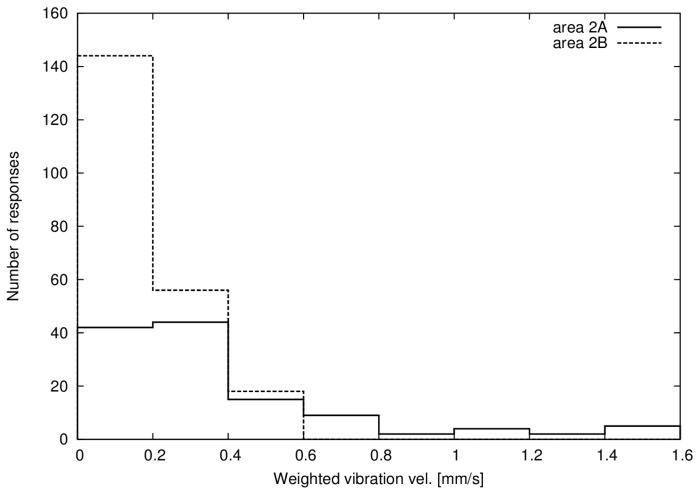
Histogram of number of responses in 0.2 mm/s intervals of weighted maximum vibration velocity for areas 2A and 2B (noise and vibration).

The results in Kungsbacka (2B) were very similar for ground and indoor vibration, and all buildings were of the same overall construction, a concrete slab with two floor levels and a wooden frame. For Alingsås (2A) the results demonstrated a much greater variation, and the buildings were also of many different construction types, with and without basement and of concrete, brick and mortar or wood construction. The amplification factor from ground to indoor vibration velocity varied between 0.7 to 5.3 in Alingsås (0.96 to 1.1 for Kungsbacka), meaning that building resonances could increase the vibration by more than five times, but the building could also reduce the vibration velocity by 30%, depending on the details of the construction. This makes it possible to predict the indoor vibration velocity with acceptable uncertainty for all respondents from the ground vibration velocity in Kungsbacka (2B), but not in Alingsås (2A). In the following study we therefore use the outdoor ground vibration during analysis, which corresponds well to indoor values for Kungsbacka, and for now accept the high uncertainty introduced in Alingsås with this approach. The number of respondents and range of vibration velocities are presented in Table 2 and Figure 2.

### 2.5. Statistical Analysis

The questionnaire data that were analyzed for this study were two adjacent annoyance questions identically worded for railway noise and vibration with an 11-point numerical scale coded as 0–10. We consider the range 5–10 to correspond to “annoyed”. Since the outcome variable annoyance is categorical and the exposure variable is continuous, we choose to use the logistic regression approach, a common approach in socio acoustic surveys [18,19,20]. The dose-response relationship is determined using logistic regression for the probability of being annoyed *P* according to

(1) $$\mathrm{logit}\left(P\right)=\beta_{0}+\beta_{1}x$$

where *x* is either the maximum weighted vibration velocity *v* or the equivalent noise level *L*_AEq,24h_ and *β*_0_, *β*_1_ are the regression coefficients. In most of the analyses below we use the logarithm of the velocity log_10_(*v*) to make it comparable to the logarithmic nature of sound pressure levels.

The function of equal annoyance was determined by setting the probability of annoyance for vibration and for noise equal to each other and solving the resulting equation. In order to estimate the confidence interval for the relation we used bias-corrected and accelerated (BC_a_) bootstrapping intervals [21,22]. Bootstrapping is a data resampling technique that can be used for approximating the sampling distribution of an estimator, when the theoretical distribution is complicated or unknown. Data is repeatedly resampled with replacement, and each sample analysed, in order to produce a distribution of values for the estimate. BC_a_ is a method that adjusts the bootstrap confidence intervals to account for bias and skewness in the distribution.

In order to compare the results presented in this article with the annoyance curves of Miedema and Oudshoorn [12] it is necessary to convert the calculated noise levels from the 24 h equivalent level *L*_AEq,24h_ to the European common noise indicator *L*_den_ according to

(2) $$L_{\mathrm{den}}=L_{\mathrm{AEq},24h}+C$$

The correction term *C* depends on the number of trains that occur at evening and night time; theoretically the correction term is zero if no traffic occurs at night or evenings, and it is 10 dB if all traffic occurs at night. For the areas here the night time traffic is relatively intense and most of the trains during night time are freight trains, which have a larger contribution to the total noise energy since they are both noisier than passenger trains and the transit times are typically longer. The correction term calculated using the Nordic method [15] is 7.4 dB for area 1 (noise only) and 7.2 dB for area 2 (noise and vibration).

In order to study the interaction between noise and vibration on the annoyance from vibration and noise we used a regression equation of the form

(3) $$\mathrm{logit}\left(P\right)=\beta_{0}+\beta_{1}L_{\mathrm{AEq},24h}+\beta_{2}\log_{10}\left(v\right)$$

where *L*_AEq,24h_ is the A-weighted equivalent noise level and *v* the vibration velocity as above. We used this equation for both annoyance from vibration and annoyance from noise for area 2 (noise and vibration).

## 3. Results

In order to estimate the exposure at which noise and vibration are equally annoying we start from dose-response relationships estimated using logistic regression between annoyance and noise/vibration. Some of the questionnaire respondents were exposed to noise but not vibration, and some were exposed to both, but none were exposed to vibration without accompanying noise. Therefore we can use area 1 (noise only) to estimate the annoyance from noise without vibration, but we must use another approach for annoyance from vibration without noise.

**Figure 3:**
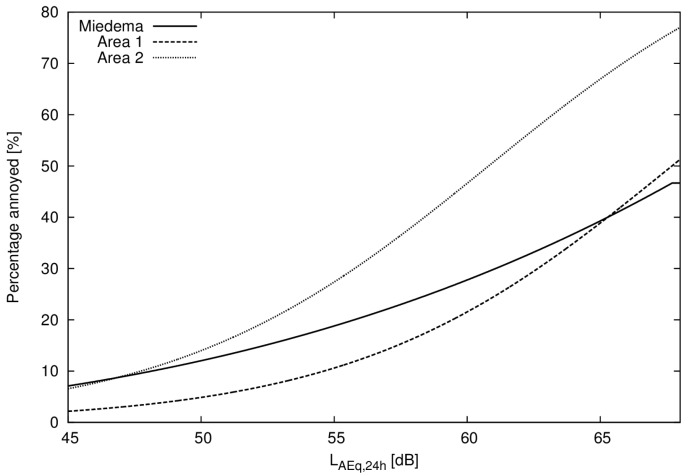
Annoyance from railway traffic noise estimated from area 1 (noise only) and area 2 (noise and vibration) compared to results from Miedema and Oudshoorn [12].

The regression results for noise annoyance as a function of noise exposure were determined separately in area 1 (noise only) and area 2 (noise and vibration) and are presented in Table 3. Compared to the annoyance curve estimated by Miedema and Oudshoorn [12] the estimated percentage of annoyed deviates for lower and for higher levels (see Figure 3). Note that the curve estimated by Miedema and Oudshoorn is a polynomial, not a logistic function.

For vibration exposure, there is not much data without simultaneous noise exposure. In Table 4, the number of responses from both areas 1 and 2 are separated into intervals. The rows correspond to the quartiles of noise exposure in area 1 (noise only) and the columns correspond to quartiles of vibration exposure in area 2 (noise and vibration). Most of the responses are along the diagonal or below it, which means that noise level and vibration velocity are related to each other but that in some cases there is little or no vibration even if there is noise present.

The most straightforward approach of separating the effects of noise and vibration is to assume that the influence of noise on annoyance from vibration is small and directly estimate it using Formula (1) in area 2 (noise and vibration); see Table 5. However, this model estimates a probability of being annoyed at zero exposure, and there is even a probability of annoyance at negative vibration velocity, which is not physically meaningful. If we instead use the base 10 logarithm of the vibration velocity in the regression equation, we get the result presented in Table 6.

**Figure 4:**
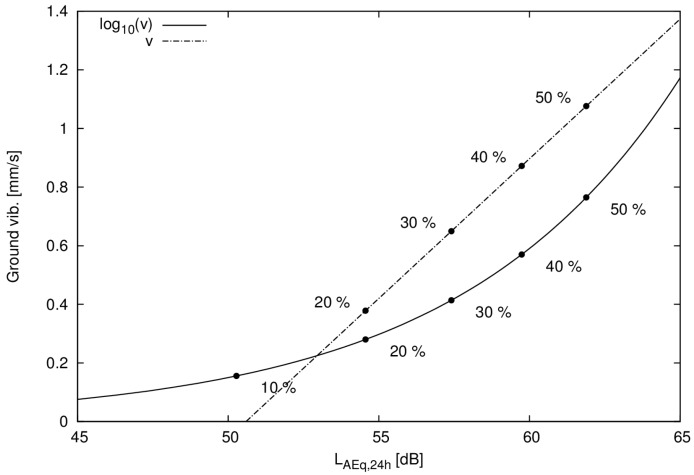
Function of equal annoyance for noise and vibration both for a linear and logarithmic model, percentages indicate probability of being annoyed either by vibration or noise.

In order to get a measure of when the annoyance from vibration equates to the annoyance from noise there are several possible approaches. The most straightforward is to put the estimated probability of being annoyed by noise equal to the probability of being annoyed by vibration in area 2. Figure 4 shows the two parametric curves that are the results of the equality of annoyance for the linear and logarithmic models of annoyance from vibration.

**Figure 5:**
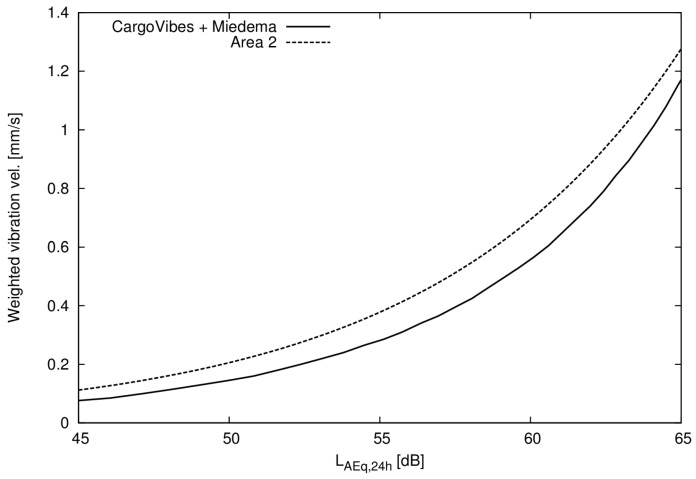
Equal annoyance curves for railway noise and vibration estimated from area 2 compared to results from CargoVibes [4] and Miedema and Oudshoorn [12].

The same approach can be used for published annoyance curves for noise [12] and vibration [4]. In order to compare this curve to our data the noise levels must be converted. We used a correction factor of 7.3 dB to convert between 24 h equivalent level and level day-evening-night, and vibration was converted using the guide available from CargoVibes [4]. Both equal annoyance curves are plotted in Figure 5.

**Figure 6:**
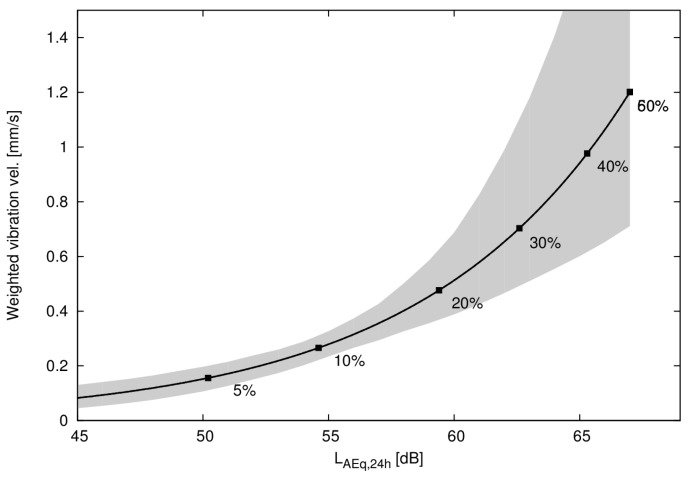
Function of equal annoyance with 95% confidence intervals estimated using bootstrapping (BC_a_). Labels indicate percentage of annoyed by vibration or noise.

The 95% confidence intervals estimated using bootstrapping with bias correction [21,22] are presented in Figure 6. Most of the data are at relatively low vibration and noise levels, which explain the rather well defined confidence intervals at these low exposure levels. For a higher annoyance probability than approximately 40%, the relation becomes more uncertain.

In order to investigate the interaction between noise and vibration in terms of annoyance we performed two regressions using Equation (3) for area 2 (noise and vibration), one for the annoyance of noise and one for vibration. The regression coefficients and *p*-values are presented in Table 7. For annoyance from noise the noise term is significant and for vibration the vibration term is significant. For the cross coefficients the *p*-value is very high for the influence of noise on annoyance from vibration, which means that higher noise levels does not modify the questionnaire response for vibration. But for the influence of vibration on annoyance from noise the opposite is true, the *p*-value is 0.022. In other words, our results indicate that annoyance from noise may be influenced by the presence of vibration, but annoyance from vibration is perhaps not influenced as much by the noise level.

## 4. Discussion

The presented analysis gives a way of comparing the annoyance from vibration and noise from railway traffic. This equal annoyance curve is useful when comparing the impact of different measures taken to reduce either noise or vibration, or when prioritizing between different traffic scenarios. It is also informative for officials when setting guidelines and target values for noise and vibration from railways. The curve calculated using questionnaire data from the TVANE areas presented here correspond well to a similar calculation using published dose response curves [4,12].

The approach used here to determine the equal annoyance curve ignores the fact that the respondents in area 1 were exposed only to noise and respondents in area 2 are exposed both to noise and vibration. If there is a strong effect of noise on annoyance from vibration the equal annoyance curves would be skewed. However, the cross terms between noise and vibration seems to be stronger for vibration causing noise annoyance than the other way around. In other words, the presence of vibration has an impact on the annoyance from noise, but the presence of noise seems to have less impact on the annoyance from vibration. These results should be interpreted with some caution however, since vibration was always accompanied by noise to some degree in this study; no test subjects were exposed to vibration only without noise.

The results are also dependent on the methodology used for the regression analysis. Assuming a relation as in Equation (3) limits the possible outcomes of the analysis. Other more advanced functional forms may give more detailed understanding at the expense of more regression coefficients to be determined. Using Equation (3) two times, once for annoyance from vibration and once for annoyance from noise, represents a simple model where vibration may influence annoyance from noise and vice versa. Note that although the model is logistic a negative coefficient represents a negative influence, i.e., a negative *β*_2_ in Equation (3) will reduce the predicted probability of being annoyed as the vibration exposure increases.

Our study is limited in terms of number of respondents and the railway traffic variation included, which could influence the relation between annoyance from noise and vibration. Vibration propagation is also strongly dependent on ground properties and in Sweden very soft clay grounds are more common than in other parts of the world. Furthermore, the building materials and construction methods vary greatly between different countries and will therefore influence the transmission of vibration from the ground and into the building.

The uncertainty in predicting the indoor vibration velocity is a major concern for all research that includes vibration from traffic as an exposure. In our study not much is known about the indoor vibration velocity or noise level of the exposed population, which is also often the case in other research areas; for example, using outdoor air pollution as a proxy for indoor air pollution.

Future research could use measured vibrations or use advanced prediction schemes for the vibration that can take as much as possible of the complexity into account, such as local ground properties and the construction details of the buildings. An alternative is to use questionnaire data to infer the exposure via questions about vibration perception, which is a way of using the exposed persons as a measurement device.

## 5. Conclusions

Based on logistic regression utilizing 862 questionnaire responses and exposure calculations of railway noise levels and vibration we have estimated an equal annoyance curve (Figure 4), a curve that describes what noise level from a railway that is equally annoying as a certain vibration velocity from a railway.

Regression results indicate that there is almost no influence of noise level on annoyance from vibration, but that the vibration velocity does influence annoyance from noise.

## Figures and Tables

**Table 1:** Daily train passages at the study sites.

Site	Total Trains	Freight Trains	Total Night-Time 22:00–06:00	Freight Night-Time 22:00–06:00
Area 1—Falköping and Töreboda	124	44	26	20
Area 2A—Alingsås	206	48	43	23
Area 2B—Kungsbacka	179	22	27	14

**Table 2:** Exposure for the questionnaire responses.

Site	Number of Responses	*L*_AEq,24h_ (dB) Mean (Min–Max) St. Dev.	Vibration Vel. (mm/s) Mean (Min–Max) St. Dev.
Area 1 (noise only)	521	52.7 (40.8–64.9) 4.1	N.A.
Area 2 (2A + 2B)	341	49.7 (41.2–63.7) 4.9	0.27 (0.10–1.50) 0.24
Area 2A (Alingsås)	123	49.8 (41.3–63.7) 5.3	0.38 (0.10–1.50) 0.34
Area 2B (Kungsbacka)	218	49.6 (41.2–61.6) 4.7	0.20 (0.10–0.49) 0.10

**Table 3:** Results from logistic regression with annoyance from noise as the outcome (area 1, noise only; and area 2, noise and vibration).

Parameter	Est.	Std. Error	z Value	*p*-Value
Area 1, noise only				
*β*_0_ (Constant)	−11.4	1.96	−5.81	<0.001
*β*_1_ (*L*_AEq,24h_)	0.168	0.036	4.73	<0.001
Area 2, noise and vibration				
*β*_0_ (Constant)	−10.2	1.56	−5.01	<0.001
*β*_1_ (*L*_AEq,24h_)	0.168	0.0297	5.66	<0.001

**Table 4:** Number of questionnaire responses and annoyance in quartiles of equivalent noise level (area 1) and maximum weighted vibration velocity (area 2).

	Number of Questionnaire Responses and Annoyance				
	Maximum Weighted Vibration Velocity *v* (mm/s)				Annoyance Noise
Equiv. SPL *L*_AEq,24h_ (dB)	≤0.14	0.14–0.19	0.19–0.31	>0.31	
≤50.4	211	57	56	17	5.6%
50.4–52.2	126	13	12	14	1.6%
52.2–54.8	138	6	23	10	9.6%
>54.8	130	0	4	45	18.6%
Annoyance vibration	4.6%	4.7%	17.0%	45.1%	

**Table 5:** Results from logistic regression with annoyance from vibration as the outcome (area 2, both noise and vibration).

Parameter	Est.	Std. Error	z Value	*p*-Value
*β*_0_ (Constant)	−2.13	0.219	−9.73	<0.001
*β*_1_ (*v*)	1.98	0.5178	3.83	<0.001

**Table 6:** Results from logistic regression with annoyance from vibration as the outcome, and with the decimal logarithm of the vibration velocity as the predictor (area 2, both noise and vibration).

Parameter	Est.	Std. Error	z Value	*p*-Value
*β*_0_ (Constant)	0.369	0.344	1.07	0.283
*β*_1_ (log_10_(*v*))	3.18	0.558	5.70	<0.001

**Table 7:** Estimated coefficients for logistic regressions of annoyance from noise and vibration in area 2 (noise and vibration).

Annoyance from	$\beta_{1}$ (*L*_AEq,24h_)	*p*-Value	$\beta_{2}$ (log_10_(*v*))	***p*** **-Value**
Noise	0.082	0.0087	2.08	0.022
Vibration	0.016	0.72	2.93	<0.001
